# Responses of a soil fungal community to severe windstorm damages in an old silver fir stand

**DOI:** 10.3389/fmicb.2023.1246874

**Published:** 2023-11-10

**Authors:** Francesco Venice, Alfredo Vizzini, Roberto Danti, Gianni Della Rocca, Antonietta Mello

**Affiliations:** ^1^Institute for Sustainable Plant Protection (IPSP) - SS Turin - National Research Council (CNR), Turin, Italy; ^2^Department of Life Sciences and System Biology, University of Turin, Turin, Italy; ^3^Institute for Sustainable Plant Protection (IPSP) - National Research Council (CNR), Sesto Fiorentino (FI), Italy

**Keywords:** fungal community ITS, forest disturbance, *Armillaria* and *Annosum* root rot, Vallombrosa forest, *Abies alba* ecosystem

## Abstract

Forests are increasingly threatened by climate change and the Anthropocene seems to have favored the emergence and adaptation of pathogens. Robust monitoring methods are required to prevent biodiversity and ecosystems losses, and this imposes the choice of bioindicators of habitat health. Fungal communities are increasingly recognized as fundamental components in nearly all natural and artificial environments, and their ecosystem services have a huge impact in maintaining and restoring the functionality of ecosystems. We coupled metabarcoding and soil analyses to infer the dynamics of a fungal community inhabiting the old silver fir stand in Vallombrosa (Italy), which is known to be afflicted by both *Armillaria* and *Annosum* root rot. The forest was affected in 2015, by a windstorm which caused a partial falling and uprooting of trees. The remaining stand, not affected by the windstorm, was used as a comparison to infer the consequences of the ecosystem disturbance. We demonstrated that the abundance of pathogens alone is not able to explain the soil fungal differences shown by the two areas. The fungal community as a whole was equally rich in the two areas, even if a reduction of the core ectomycorrhizal mycobiome was observed in the wind-damaged area, accompanied by the increase of wood saprotrophs and arbuscular mycorrhizas. We hypothesize a reshaping of the fungal community and a potentially ongoing re-generation of its functionalities. Our hypothesis is driven by the evidence that key symbiotic, endophytic, and saprotrophic guilds are still present and diversified in the wind-damaged area, and that dominance of single taxa or biodiversity loss was not observed from a mycological point of view. With the present study, we aim at providing evidence that fungal communities are fundamental for the monitoring and the conservation of threatened forest ecosystems.

## 1. Introduction

Forest disturbances associated with extreme events and natural disasters are important drivers of forest ecosystem development. Furthermore, they are expected to continuously increase in intensity, quantity, and frequency in the coming years, seriously threatening the world’s forest (Masiero et al., 2019). Strong winds, one of the major natural disturbances for European forests (Seidl et al., 2011; Forzieri et al., 2020), have intensified over the last decades globally and their natural and socio-economic consequences can be especially critical (Romagnoli et al., 2023). Climate change will make these destructive windstorm events more frequent and more intense with damage and destruction of thousands of hectares of forests (uprooting and breaking trees) and millions of cubic meters of timber lost (Patacca et al., 2023). Many destructive severe windstorm were recorded in Europe in recent years: windstorm Lothar (1999) caused the loss of 165 million m^3^ of timber in France, Germany, and Switzerland; windstorm Gudrun (2005) 75 million m^3^ in Sweden; windstorm Kyrill (2007) 49 million m^3^ in Germany and the Czech Republic; windstorms Klaus (2009) in France and Xynthia (2010) in Spain a total of 45 million m^3^; windstorm Vaia (2018) in Italy 8.5 million m^3^ (Forzieri et al., 2020).

An extreme wind event, also affected in 2015 the Vallombrosa forest, one of the most famous and studied forests in Italy (northern Apennines), the birthplace of the Italian Forestry School in the late 19th century, and today a teaching forest for students of Forestry and Environmental Sciences at the University of Florence. On the night of March 5, 2015, between 15 and 20 thousand trees (about 50 ha) were blown down in the forest by a hurricane, with wind gusts reaching 150–160 kilometers per hour (Chirici et al., 2019; Dálya et al., 2019).

The forest of Vallombrosa, today a Biogenetic State Nature Reserve and a Natura 2000 Site, is also famous because the monks of the Vallombrosa Abbey (Benedictine order) in the XVII century began a centuries-old tradition, widespread to much of Central Europe, of growing pure and coetaneous silver fir (*Abies alba* Mill.) stand that still characterizes part of the forest landscape (Ciancio and Nocentini, 2011) that is now managed by the State body Carabinieri Forestali.

During an investigation in this forest Farina et al. (1990) observed the massive presence of *Heterobasidion abietinum,* which is among the most destructive forest pathogens commonly associated with European silver fir, and other species of the genus Abies (Gonthier and Thor, 2013). *H. abietinum* causes root rot and decay of the stem, which typically leads to decrease of the tree stability and uprooting under certain stressful conditions (Honkaniemi et al., 2017). A severe windstorm occurred in April 2015 at the Nature Reserve of Vallombrosa and, as a consequence, about 50 ha of forest were destroyed. In an investigation after the severe windstorm damage in the forest of Vallombrosa, Dálya et al. (2019) assessed the distribution of *Heterobasidion abietinum*, the presence of which had been already reported by Farina et al. (1990), and *Armillaria* spp. which had only been observed sporadically and are among the most destructive forest pathogens in the world. In this research, *H. abietinum* presence was confirmed and extended at two new localities at upward elevation, an occurrence probably favored by climate change. Four species of *Armillaria* (*A. cepistipes*, *A. ostoyae*, *A. gallica*, *A. mellea*) were found in the area of the Nature Reserve of Vallombrosa, among which the most frequent species was *A. cepistipes*, followed by *A. ostoyae*, which was often detected just in soil samples from plots cultivated with conifers (Dálya et al., 2019).

Both *H. abietinum* and *A. ostoyae* are known as fearsome silver fir pathogens, typically show higher incidence in artificial stands derived from monospecific and coetaneous plantations exposed to climatic stress (La Porta et al., 2008). An additive effect to the high presence of *H. abietinum* is also the ‘history’ probably related to the development of the existing microbial community. In fact, a higher presence of this pathogen has been observed in former cropland or former pastureland than in forest soils (Puddu et al., 2003), condition very frequent in some areas of the Vallombrosa forest (Galipò et al., 2017; Caramalli et al., 2020).

The role played by the telluric pathogens described above in the event that led to the uprooting of many silver firs in Vallombrosa is still much debated. In areas with such a disturbance the status and resilience of the soil microbial community, which is fundamental for the forest ecosystems functioning, are little known. Identifying the drivers of microbial community stability is crucial for predicting community response to disturbance. These drivers are defined as ‘keystone taxa’, capable of influencing the community structure through strong interactions with the environment or with other members of the microbiome in co-occurrence networks (Trivedi et al., 2020). Among forest microorganisms, fungi have several key roles as decomposers of the organic matter and as plant symbionts, and support numerous ecosystem services, acting as a crucial tool for the adaptation of forests to climate change (Van der Heijden et al., 2015; Mello and Balestrini, 2018; Sapsford et al., 2021). Recent metabarcoding studies targeting the rDNA Internal Transcribed Spacer (ITS) have been shown to be the best tools to describe fungal communities (Nilsson et al., 2019) and to identify keystone taxa.

In order to assess the soil microbial community in the silver fir stand at ‘Metato’ (Natural Reserve of Vallombrosa, Reggello, Florence), affected by *H. abietinum* and *Armillaria* spp. and characterized by both presence of undamaged and wind-damaged areas (stumps), we profiled soil fungal communities by using metabarcoding, and combined sequencing results with soil physico-chemical parameters. The objectives of this work were the following: to investigate relations in the soil fungal community associated with *A. alba* undamaged and wind-damaged areas. We hypothesize that an ecological succession has occurred since the windstorm event, and hence that each condition is associated to a peculiar functional guild: for instance *A. alba* undamaged trees associate with ectomycorrhizal fungi, while stumps and the surrounding vegetation cover associate with Mortierellomycetes, arbuscular mycorrhizal fungi, and lignicolous fungi.

## 2. Materials and methods

### 2.1. Description of the site, soil sampling and soil physico-chemical analyses

**Figure 1:**
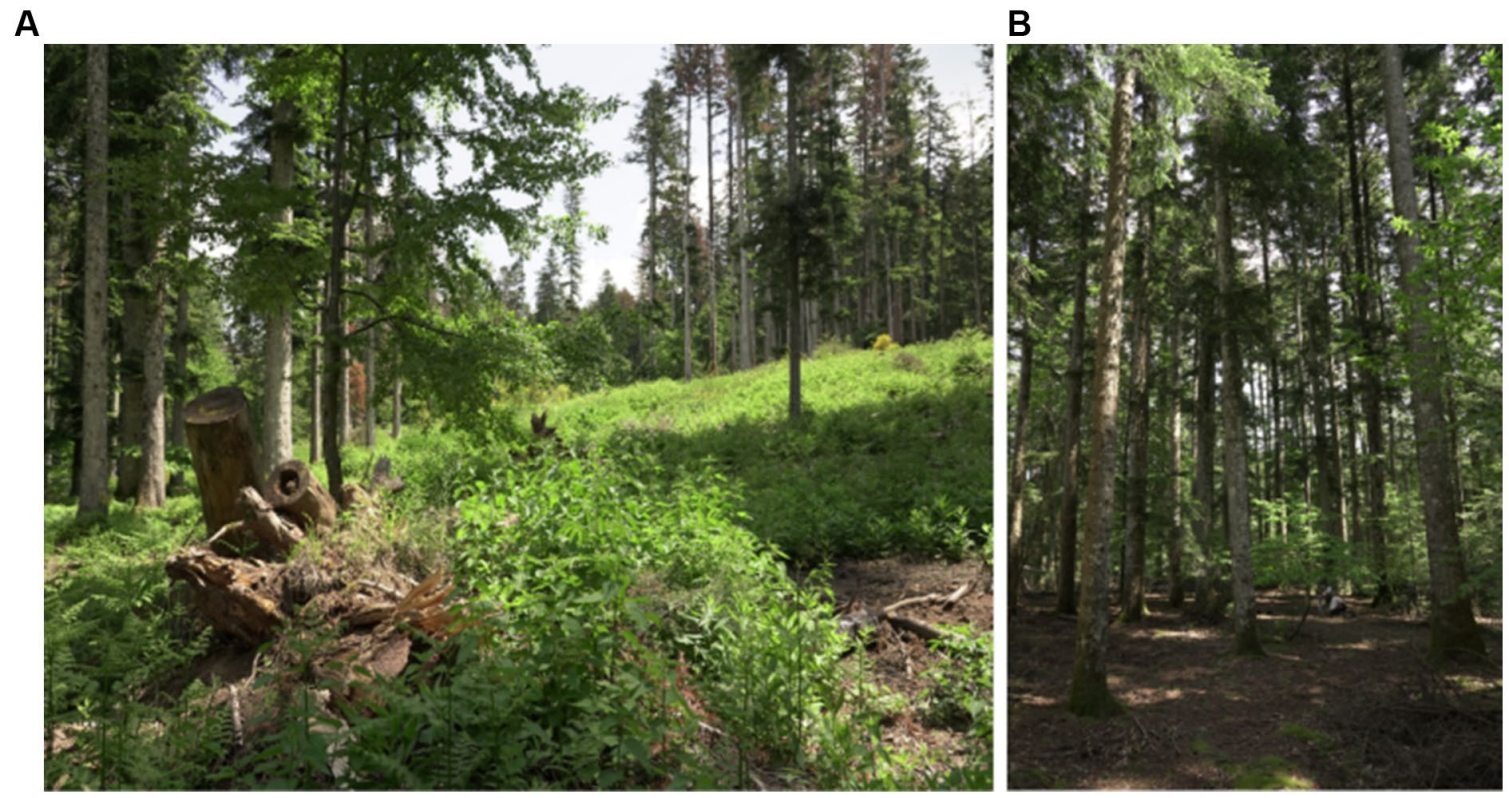
Wind-damaged **(A)** and undamaged **(B)** areas at the “Metato” site. The gaps in the forest caused by the windstorm allowed an increase of grasses and undergrowth. By contrast, the dense canopy cover in the undamaged area impedes this phenomenon.

The investigated mature and pure silver fir stand of approximately 11 ha called ‘Metato’, a stone hut in a chestnut grove, for drying chestnuts, which, piled on mats, are subjected to moderate heat, known as a former farm until the late 1800s (Galipò et al., 2017), is located in the Vallombrosa Biogenic reserve (Tuscan Apennines in Florence district, Italy). The climate is characterized by a mean annual temperature of 9.8°C and a mean annual precipitation of 1,275 mm (thermopluviometric station of Vallombrosa, 980 m a.s.l.; Dálya et al., 2019). The trees in the area are known to be affected by *Annosum* and *Armillaria* root rot, which may have been a possible contributing factor of tree uprooting as a consequence of a windstorm in 2015, therefore segmenting the forest in two areas ‘undamaged’ and ‘wind-damaged’ (Figure 1) where trees were felled down by the hurricane and an extensive gap in forest cover was created. According to the pedological map of Tuscany Region, the soil is mainly classified as partially humic dystrudepts, coarse-loamy, mixed, mesic.

Soil collection was carried out in two areas located at least 20 m apart from each other: one of 59,400 m^2^ characterized by undamaged trees and the other of 50,600 m^2^ by wind-damaged stumps. Sampling was done under six silver fir trees and six stumps: five soil cores (200 cm^3^ each) of topsoil from underneath the litter layer were collected around two meters from each tree or stumps in opposite directions, and at least two meters from each other to avoid sampling of the same genet. Each biological replicate consisted in a pool of five soil samples (coarse roots and stones were removed), resulting in a composite soil sample for each tree. A subset of each sieved, composite soil (500–750 g; mesh size 1 mm) was used for physico-chemical analyses, and another for microbial community analysis by metabarcoding.

Soil samples were heated overnight at 105°C to determine their water content (ISO 11465:1993), while sieving and sedimentation were used for texture evaluation (ISO/FDIS 11277). A potentiometric evaluation was performed to determine the pH of each sample, using a mix of one part air-dried soil and 2.5 parts deionized water (1:2.5; samples were left to equilibrate overnight). BaCl_2_-based compulsive exchange method (ISO 11260:2018) was used to quantify cation-exchange capacity (CEC) and soil exchange acidity. Exchangeable cations K^+^, Na^+^, Mg^2+^, Ca^2+^ were determined by a 1100B Atomic Absorption Spectrometer (Perkin Elmer, United Kingdom). Total C and N were determined using a NA 1500 CHNS Analyzer (Carlo Erba, Italy). Phosphate was extracted from soil samples using HCl and NH_4_F, therefore removing acid-soluble P forms (Bray and Kurtz, 1945). The R package ggridges v0.5.3^1^ was used for density plots showing the results of all the physico-chemical analyses. Significant differences between undamaged and wind-damaged samples were determined with Kruskal-Wallis test in R at a 0.05 value of *p* threshold.

### 2.2. DNA extraction, sequencing, and OTU table generation

Twelve silver fir trees and stumps (6 vs. 6) were considered for the analysis. Three DNA extractions (technical replicates) have been performed for each composite soil sample with the FastDNATM SPIN Kit for Soil (MP Biomedicals, Europe). A total of 36 DNA samples were therefore diluted 1:10 (5–10 ng/μl) and amplified with a nested PCR approach targeting the Internal Transcribed Spacer 2 (ITS2) (Nilsson et al., 2019). A first amplification was done with the ITS1/ITS4 primers couple (5’-TCCGTAGGTGAACCTGCGG-3′ and 5’TCCTCCGCTTATTGATATGC-3′, respectively), for 25 cycles at 52°C annealing temperature. The PCR products were again amplified in a second round with the ITS9f/ITS4r primers (5’-GAACGCAGCRAAIIGYGA-3′; 5’-TCCTSCGCTTATTGATATGC-3′) to which Illumina adapters have been added (TCGTCGGCAGCGTCAGATGTGTATAAGAGACAG and GTCTCGTGGGCTCGGAGATGTGTATAAGAGACAG, respectively), for 35 cycles at 54°C annealing temperature. The libraries have been sequenced at IGA technologies (Italy) using Illumina MiSeqTM with a paired-end strategy (2×300 bp, NexteraXT index kit) producing 10 million reads in output. FastQC (Andrews et al., 2012) was used for quality assessment of the libraries, and primers were removed with Cutadapt v3.4 (Martin, 2011). The trimmed libraries were processed within the DADA2 pipeline v1.18.0 (Callahan et al., 2016). Quality trimming was achieved with the “filterAndTrim “function [“maxEE(2,7)”], setting a minimum length threshold of 165 bp for trimmed reads. Error models were produced through the evaluation of 1E8 bases, and errors were removed from de-replicated reads using pseudo-pooling with the “dada” function. Merged forward and reverse reads were subjected to *de novo* and reference-based chimera screening using DADA2 and VSEARCH v2.17.0 (Rognes et al., 2016), respectively. For the latter case, the UNITE v8.3 fungal ITS database (Nilsson et al., 2019) was used as reference. ITS2 sequences were extracted from the dataset with ITSX v1.1.3 (Bengtsson-Palme et al., 2013). The dataset was then processed with QIIME2 v2020.11 (Bolyen et al., 2019) clustering the extracted sequences into OTUs at 97% identity.

### 2.3. Taxonomy assignment and validation

Centroids were used for a BLASTn v2.11 (Camacho et al., 2009) search against the nt database. Taxonomy annotation was achieved with the “Assign-Taxonomy-with-BLAST” scripts,^2^ using the following criterion: a maximum of 10 BLAST hits were considered if they fell in a 0.5% identity interval based on the best BLAST hit. For example, if an OTU had 100% identity with its best BLAST hit, other BLAST hits were considered only if they had at least 95% identity with the OTU. Species-level annotations were considered valid based on a 97% identity threshold. Family- or phylum-level annotations were instead assigned based on 80 and 75% identity thresholds, respectively. OTUs with divergent taxonomy assignment were resolved with a phylogenetic approach. For each of these OTUs, the whole sequence cluster and its best BLAST hits were aligned with MUSCLE v3.8.31 (Edgar, 2004). The alignments were processed with Gblocks v0.91b (Talavera and Castresana, 2007) with relaxed parameters, and phylogenetic trees were produced with FastTree v2.1 (Price et al., 2010). Trees were inspected to manually curate the inferred phylogenetic relationships, allowing to deal with “unidentified” taxonomies which are abundant in the NT, and that were the most common cause of taxonomic inconsistency in OTUs. After taxonomic assignment, OTUs that did not belong to the Fungal Kingdom were discarded. Functional guilds were assigned to each OTU with FungalTraits v0.0.3 (Põlme et al., 2020), and manually curated by an expert mycologist.

### 2.4. Core community identification, alpha and beta diversity measures, and correlation analyses

Prior to fungal community analyses, the OTUs abundances were normalized using median library size, according to the protocol proposed in the phyloseq v3.12 package (McMurdie and Holmes, 2013). Taxonomy plots were generated combining the “subset_taxa” phyloseq function and the “ggstripchart” function of the ggpubr v0.4.0 package (Kassambara, 2020). The R packages phyloseq, microbiome v1.13.3 (Lahti and Shetty, 2012), and ggplot2 v3.3.3 (Wickham, 2009) were used for the core community analysis in accordance with Shetty et al. (2017). In this case, the ratio between the abundance of each family and the abundances of all other taxa (relative abundance) was plotted. Taxa with a relative abundance <0.01%, or that were above this threshold in less than 60% of samples were discarded. Statistical significance of core relative abundances between healthy and diseased samples was calculated with Kruskall-Wallis test (*p* < 0.05). Alpha- and beta-diversity values were computed with phyloseq and QIIME2, respectively. The correlation between the abundance of *Armillaria* and *Heterobasidion* pathogens, and soil physico-chemical parameters was calculated with the base “cor” R function and plotted with ggplot2. The correlation threshold for the analysis was set to 70%.

### 2.5. Differential taxa abundances

DESeq2 v1.30.1 (Love et al., 2014) was used for the final estimation of differentially abundant OTUs, starting from raw abundances. The DESeq2 pipeline involved physico-chemical soil parameters measured for each sample, and the included variables were selected as follows: at first, to avoid multicollinearity among covariates, the correlation between these variables was calculated with the “cor” base R function, and a 70% threshold was set to discard highly correlated variables. The correlation plot was generated with the pheatmap package v1.0.12 (Kolde, 2019). A Redundancy Analysis (RDA) plot was then produced using the RAM 1.2.1.7 package (Chen et al., 2018) to screen for variables influencing the distribution of samples. The RDA analysis was coupled with a variance partitioning analysis in Vegan v2.5–7 (Oksanen et al., 2020). Statistical support was calculated with ANOVA (*p* < 0.05). Uncorrelated variables that significantly impacted the variance in the dataset were finally used as covariates in the DESeq2 design formula. The dataset was further filtered to include only OTUs that had a summed abundance >10. DESeq2 was run with the “betaprior” option, and the “contrast” function has been used to calculate differential abundances (the wind-damaged condition was used as numerator), at an adjusted value of *p* threshold of 0.05. The results were plotted using metacoder v0.3.4 (Foster et al., 2017).

## 3. Results

### 3.1. Soil analyses and taxonomy overview

**Figure 2:**
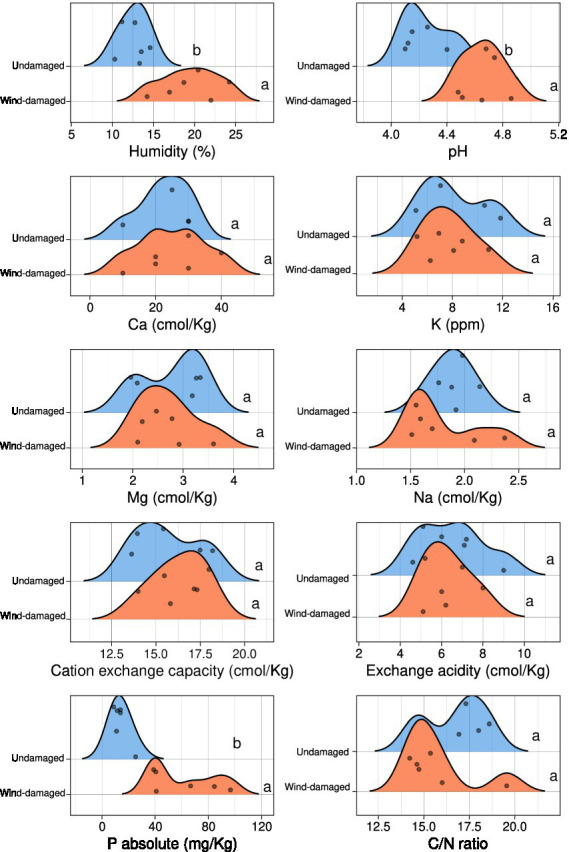
Physico-chemical characteristics of soils collected near the same stumps and undamaged trees that were selected for metabarcoding. For each measurement, the density plots show the number of samples (represented by dots) having a specific value in the wind-damaged or undamaged area. Statistical significance is shown in letters (Kruskall-Wallis test 0.05 threshold).

**Figure 3:**
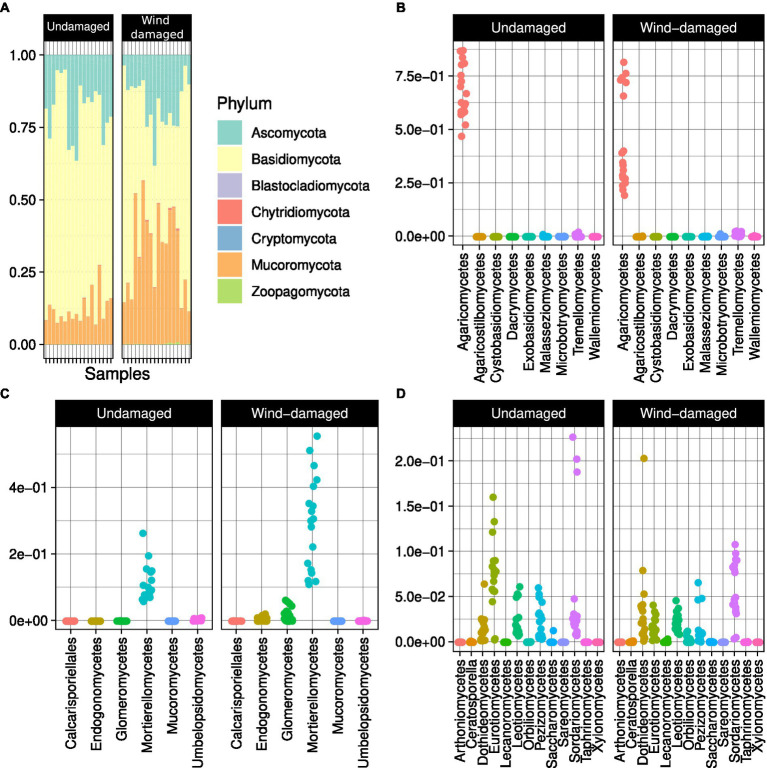
Overall composition of the fungal community in Vallombrosa. **(A)** Basidiomycota were predominant overall in terms of relative abundance (i.e., their abundance over the abundance of all OTUs). The classes Agaricomycetes **(B)** and Mortierellomycetes **(C)** were predominant in Basidiomycota and Mucoromycota, respectively, while Ascomycota **(D)** had a more even class distribution. Dots in **(B)**, **(C)**, and **(D)** represent samples and are distributed according to the relative abundances of each class (y axis).

The soil samples obtained from the surroundings of stumps from the wind-damaged area had higher humidity, pH, and phosphate content (Figure 2). The other analyzed soil parameters did not differ between the two conditions (wind-damaged and undamaged). The final OTU table obtained from the fungal sequences extracted from the whole silver fir stand soil is shown in Supplementary Table S1. The composition of the fungal community in the two areas showed the overall prevalence of the phylum Basidiomycota (Figure 3). However, this dominance was attenuated in soils sampled in the wind-damaged area, where Mucoromycota showed quite high relative abundance. Inside the Basidiomycota phylum, Agaricomycota were the dominant class in both areas. Mucoromycota were mostly represented by Mortierellomycetes in the undamaged area; by contrast, Glomeromycetes and Endogonomycetes contributed to the increase in Mucoromycota observed in the wind-damaged area. Ascomycota were well-represented in both sites, with an even distribution of abundances in Dothideomycetes, Eurotiomycetes, Pezizomycetes, and Sordariomycetes.

### 3.2. Pathogen detection and core community analysis

At first, we manually searched for the presence of the two root-rot agents, *Armillaria* and *Heterobasidion*, in the dataset: both were present irrespective of the undamaged and wind-damaged conditions, even if absent from several samples (Supplementary Table S1). We could not obtain a species-level classification for the two OTUs corresponding to *Armillaria* and *Heterobasidion* but, according to literature data, the most abundant species found were *A. ostoyae* and *H. abietinum* (Dálya et al., 2019). We also checked whether the presence of *Armillaria* and *Heterobasidion* was correlated with any of the measured soil properties (Figure 2), irrespective of the sampling area, but found that none of the measured parameters influenced the abundance of the pathogens (Supplementary Figures S1).

**Figure 4:**
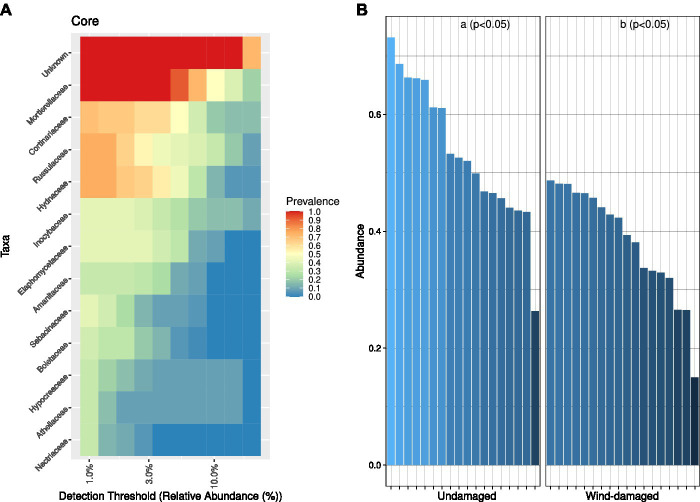
Core fungal community analysis. **(A)** Core community composition in terms of predominance, i.e., the fraction of samples (1 = all samples and 0 = no sample) in which a specific family had at least the relative abundance defined on the x axis. For example, based on the representation, Mortierellaceae were present at 3% relative abundance in all samples, and at 10% ~50% of samples. **(B)** For each sample and sub-area, we divided the abundances of OTUs representing the core mycobiome, by the abundance of all OTUs (relative abundance). The core fungal community was more abundant in the undamaged area (Kruskall-Wallis test at *p* < 0.05).

At the family-level, the core microbiome of the whole site was represented, after unknown families, by Mortierellaceae (with at least 50% relative abundance in 10% of the samples) followed by families such as Cortinariaceae (mostly ectomychorrhizal), Russulaceae, and Hydnaceae (Mycorrhizal; Figure 4A). This core community was overall less represented in soil samples from the wind-damaged area (Figure 4B).

### 3.3. Alpha diversity analyses

**Figure 5:**
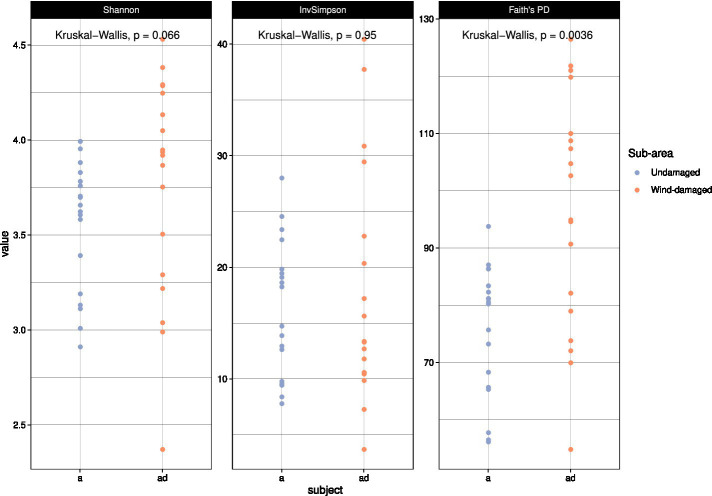
Alpha diversity calculations. The color codes represent undamaged (a) vs wind-damaged (ad) samples, and are placed on the vertical axis based on their alpha diversity values according to each index. Significance values were calculated with ANOVA, at *p* < 0.05.

With alpha diversity calculations, we aimed at investigating a potential impact on fungal biodiversity in soil as a consequence of the fall of the trees, and of the subsequent creation of gaps in the forest. At first, we used all the taxa in the dataset to obtain different alpha diversity measures (Figure 5). Only Faith’s phylogenetic diversity (PD) index allowed us to detect a higher biodiversity in the wind-damaged area. Simpson and Shannon indices are preferentially used to calculate dominance and diversity, respectively, while Faith’s PD has been frequently used to measure the diversity of fungal successions (Zhang et al., 2018; Matsuoka et al., 2019; Adamo et al., 2021). The latter is fitted on a phylogenetic backbone, and it does not consider OTUs individually, but also as a function of their phylogenetic distance. Indeed, the index is based on the sum of all branch lengths observed in the OTUs tree. The generation of OTUs (and their number) is biased by the low taxonomic resolution typical of fungal amplicons, which makes OTUs richness a weak measure for fungal biodiversity (Song, 2023). By contrast, phylogenetic reconstructions might help mitigating such phenomenon: indeed, two OTUs that were erroneously considered as different taxonomic entities would be very closely related in a phylogenetic tree, lowering their overall impact on biodiversity measures. PD has been indeed noted to be more sensitive in investigating biodiversity data (Armstrong et al., 2021). Therefore, we decided to use this index for further analyses, even if it introduces an evolutionary point of view that does not fit the present investigation.

To further investigate the impact of fall of the trees on the alpha diversity of specific functional guilds, we measured the same alpha diversity indices on the ectomycorrhizal and wood-decay communities separately (Supplementary Figures S2, S3). Faith’s PD was higher for the wood-decay community in the wind-damaged area; by contrast, all alpha diversity indices indicated a strong reduction of the ectomycorrhizal diversity as a consequence of fall of the trees.

### 3.4. Beta diversity analysis and differential taxa abundance

**Figure 6:**
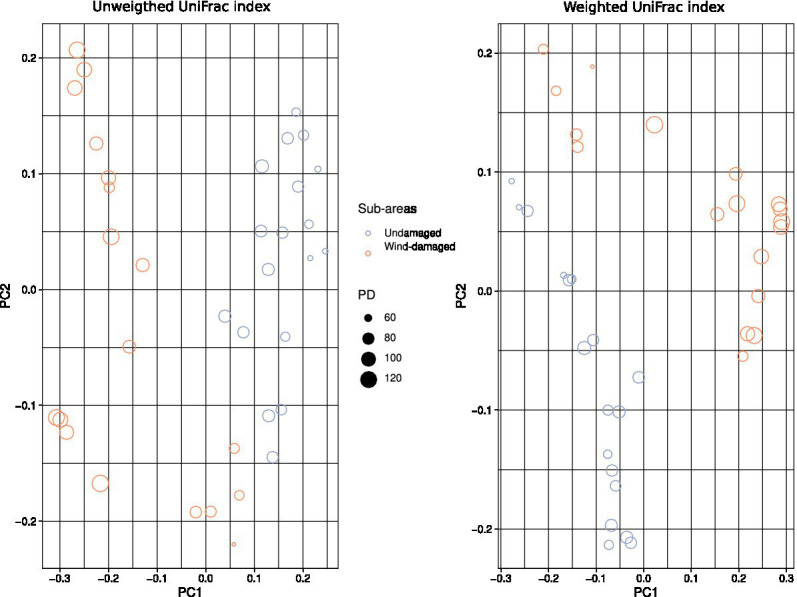
Beta diversity calculations. The color and shapes of the dots represent undamaged vs. wind-damaged samples, and both weighted and unweighted UniFrac indices were calculated. The size of each dot (sample) is related to the Faith’s PD of that sample. Both indices highlight a separation of the two areas in terms of mycobiota composition.

To further highlight compositional differences of the mycobiota between soils sampled in the wind-damaged and undamaged area, we used both unweighted (phylogeny-based) and weighted (which further adds abundance data to the phylogeny-based method) UniFrac indices (Lozupone and Knight, 2005). In both cases beta diversity revealed a clear separation between soils from the wind-damaged area *vs* those sampled in the undamaged area (Figure 6).

**Figure 7:**
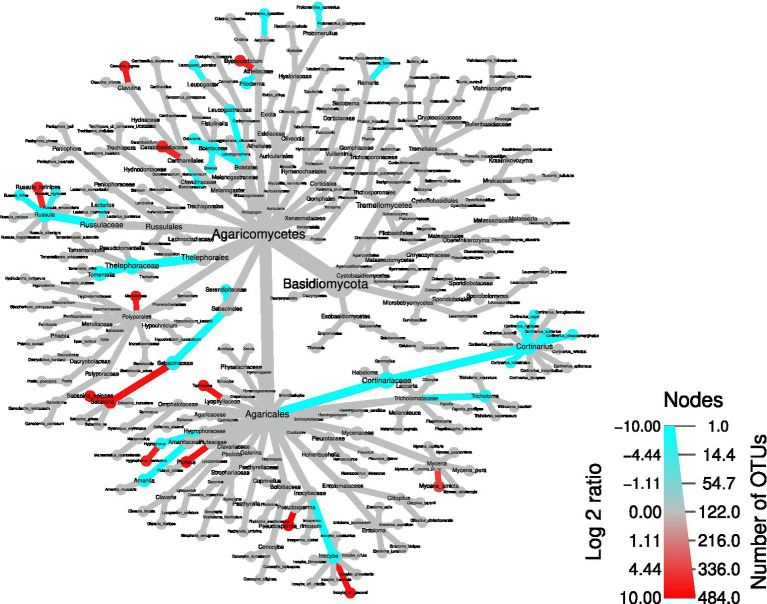
Differential abundance tree showing differentially-abundant Basidiomycota in the wind-damaged *vs* undamaged areas. Red color for nodes and edges indicates over-representation in the wind-damaged condition, while blue indicates the opposite. The color intensities were calculated by summing all log2 fold changes of OTUs gathered at each node and branch.

Next, to identify the OTUs driving these differences, a differential taxa abundance analysis was performed. Starting from the physico-chemical soil parameters that significantly differed between the two areas (Figure 2), we checked whether some of them influenced the ordination of the different samples. We removed pH, which was highly correlated with P content and humidity (Supplementary Figures S4): the choice of removing pH was due to the fact that P content and humidity were not significantly correlated, and therefore this allowed to remove only one variable. An RDA plot showed that exchange acidity, C/N ratio, cation exchange capacity, and humidity might have an influence on taxa composition (Supplementary Figures S5). Therefore, we included the above-mentioned soil parameters in the statistical model to infer differential taxa abundances. Confirming the overall screening of the OTU table, *Armillaria* and *Heterobasidion* OTUs were not among the differentially abundant taxa (Figure 7). By contrast, several dead wood-associated fungi such as *Pluteus*, *Mycena*, *Byssocorticium,* and Meripilaceae were more abundant in the area affected by falling of the trees, whereas ectomycorrhizal fungi such as *Amanita,* Boletaceae, *Cortinarius*, *Lactarius*, *Leucogaster*, *Tomentella*, and *Tricholoma* mostly underwent a depletion in such area. By contrast, *Hygrophorus*, *Pseudosperma*, *Sebacina* were more abundant in the wind-damaged area, compared to the undamaged one. In addition, *Tephrocybe* was more abundant in the wind-damaged area. Among Ascomycota, *Trichoderma* was more abundant in the wind-damaged area, while the ectomycorrhizal *Otidea*, *Tuber*, and *Trichophaea* were depleted (Supplementary Figures S6). Finally, the Arbuscular Mycorrhizal (AM) fungus *Paraglomus laccatum* and Endogonales, preferentially associated with herbaceous plants or hornworts and liverworts (Van der Heijden et al., 2015), were more abundant in the wind-damaged area (Supplementary Figures S7).

## 4. Discussion

### 4.1. Are root rot pathogens responsible for habitat disruption in Vallombrosa?

The effort of the scientific community in understanding the weakening of forests led to the conclusions that (a) the fitness of major plant pathogens increased in the Antrophocene, mainly due to climate change (Stenlid et al., 2011; Hessenauer et al., 2021), (b) the presence of these pathosystems is deeply integrated in habitat ecology, eventually contributing to biodiversity shifts (Winder and Shamoun, 2006; Gomdola et al., 2022), and (c) extreme meteorological events due to climate change are main contributors in habitat destruction. If root rot pathogens were the only determinants of tree damages, then we hypothesize that a higher abundance of *Armillaria* and *Heterobasidion* should have been detected in the wind-damaged area, a situation that we did not observe. In addition, the investigated site (Metato) is a silver fir stand whose origin dates back to the end of XIX century; the presence of *Armillaria* and *Heterobasidion* spp. was reported in this artificial and coetaneous stand since the early 1900s, while the extreme event that led to falling of the trees and uprooting in a large part of the site took place in 2015 (Dálya et al., 2019). Therefore, the history of co-existence between these pathogens and the residing tree is long, and the wind damages should likely have manifested earlier. Indeed, the incidence of both diseases is known to be extremely severe in pure and coetaneous stands planted in former agricultural land and in localities where summer drought is exacerbated by climate change (Puddu et al., 2003) as in this case.

We observed a depletion of several ectomycorrhizal taxa in the wind-damaged area, while the same taxa were abundant in the undamaged area. This result leads to the quandary of whether such depletion contributed to the localized wind damages (due to a loss of fitness of the host trees), or whether certain taxa were depleted because of the disappearance of their hosts after the fall of the trees. Evidence collected worldwide agrees that ectomycorrhizal fungi can increase forest resistance to abiotic and biotic stressors, such as fungal pathogens (Suz et al., 2015; Anthony et al., 2022; Authier et al., 2022). However, ectomycorrhizal communities are strongly impacted by climate change (Fernandez et al., 2023), and this could have been one cause of the decline of tree health in Vallombrosa as well. We therefore suggest that a depletion of ectomycorrhizal taxa preceded the observed damages. At the same time, different soil properties such as pH, humidity, and C/N content were altered in the wind-damaged area. Other authors have suggested that soil characteristics can greatly boost the spreading of *Armillaria* and *Heterobasidion* (Singh, 1983; Kubiak et al., 2017; Bruna et al., 2019; Piri et al., 2021). We found no correlation between soil parameters and the abundance of *Armillaria* or *Heterobasidion:* it is possible that, while not altering pathogen abundances, these potential abiotic stressors may have a negative impact on tree fitness and ectomycorrhizal communities. Finally, it is likely that parameters such as C/N ratio and mineral content are directly influenced by the increased amount of dead plant material accumulated after the falling of the trees.

### 4.2. Ectomycorrhizal *Agaricomycetes* and *Mortierellomycetes* as primary components of the mycobiota

Several authors have previously investigated the prominent role of Basidiomycota as biomarkers in forests (Richard et al., 2011; Ruiz Gómez et al., 2019; Venice et al., 2021). Since forests are increasingly threatened by extreme meteorological events due to climate change, studying the impact of these fungi on plant health is crucial for the conservation of biodiversity and for forest management. It has been demonstrated that ectomycorrhizal communities are sensible to habitat fragmentation caused by fires and windstorms (Sapsford et al., 2020; Idbella et al., 2023), even if there is evidence that, despite severe disturbances, the ectomycorrhizal network is affected in its composition but not disrupted (Veselá et al., 2021). The investigated site makes no exception, and a key result in our analyses was that, besides an overall reduction, the ectomycorrhizal community is still detectable in the wind-damaged area, and specific genera are core components of the “Metato” mycobiome. Ectomycorrhizal Ascomycota were also present but, however, were relatively less abundant in the investigated soils. Overall, this diversity and specificity may lead to the hypothesis that an ectomycorrhizal succession is supporting habitat restoration in Vallombrosa. Ectomycorrhizal fungi have different hyphal exploration types that vary from long-range to contact-range. It is possible that, thanks to a long-range hyphal exploration type, they maintain symbiotic associations with living trees in the undamaged area, which is close to the wind-damaged one, while extending their mycelial network in the proximity of fallen trees.

*Mortierella* is a ubiquitous group of soil saprotrophs and endophytes, and is often found as key component of conifer forests mycobiota (Allmér et al., 2006; Wagner et al., 2013; Mikryukov et al., 2021), even if its prominent role was also highlighted in other forests (Venice et al., 2021; Kalntremtziou et al., 2023). The abundance of several, specific *Mortierella* (and higher taxonomic ranks) indicates a major contribution of this fungal group in maintaining the “Metato” ecosystem functioning. Indeed, the presence of some species from this clade was preferentially observed in “sheltering” dead conifer wood (Mikryukov et al., 2021) while others were preferentially found as root endophytes (Summerbell, 2005), pointing out to different fundamental environmental services.

### 4.3. A biodiversity succession, rather than a reduction, characterizes the wind-damaged area

Our analyses indicate that fungal diversity is maintained also in the wind-damaged area By splitting the dataset, focusing the analysis on either ectomycorrhizal or wood-decay taxa, we demonstrated that tree symbionts are indeed depleted in the wind-damaged area, while wood-decayers are likely sustaining alpha diversity in this area. As for concerns the contrasting results obtained with different alpha diversity indices, a possible explanation could reside in the fact that PD is an evolutionary measure that evaluates the phylogenetic distance between the amplicon sequences of each sample. However, if PD shows significant differences while other diversity indices do not, this may be due to the history of the species library, according to the species pool hypothesis: indeed, biodiversity might not only be related with environmental or ecological factors, as it can be strongly limited by the regional species pool. In this context, Shannon and Simpson would be more appropriate indices, and they should not be overlooked also in the present study. However, irrespective of the fact that alpha diversity is equal or lower in the undamaged area, compared to the wind-damaged area, we hypothesize that the whole fungal community in the undamaged area is well-established and specialized. Therefore, redundancy in its taxonomic and functional composition might be expected. By contrast we advocate that, after a relatively recent perturbation, the wind-damaged area might constitute a less-established, re-generating habitat with a larger quantity of dead plant material and a higher degree of invasiveness by both saprotrophs and symbiotrophs. This hypothesis is also in line with the beta diversity results, which demonstrate a different composition of the communities detected with both weighted and unweighted UniFrac indices. For example, the wind-damaged area showed a strong increase in taxa such as *Mycena*, *Byssocorticium*, and *Pluteus*, all able to feed on dead wood, together with the presence of *Tephrocybe*, that grows preferentially in re-generating forest soils after disturbances that include wildfires and chemical treatments (Yamanaka, 2001; Ratkowsky and Gates, 2009; Pulido-Chavez et al., 2023). Finally, we found that the falling of the trees was associated with a higher colonization of soil by AM fungi and Endogonales. This is in line with the fact that tree falling, and the consequent absence of crown (Figure 1), allowed the growth of many grasses and hornworts, which are the preferred hosts for these fungi (Van der Heijden et al., 2015; Chang et al., 2019). As already observed for other forests (Song et al., 2020), the plant succession in the wind-damaged area could stimulate a parallel microbial succession, with rapid replacement of taxa with increasing adaptability to the rhizosphere.

## 5. Conclusion

In this study, we investigated a portion of a mature silver fir stand that was subjected to trees uprooting after a windstorm and compared it with a close area which was not visibly damaged by the storm. Due to the proximity of the two areas, we exclude the possibility that microclimatic factors drove the differentiation of the two sites in terms of damages caused by meteorological events. Our investigation was also driven by the evidence that the whole site is affected by the root rot agents *A. ostoyae and H. abietinum*, which we excluded as the only cause for the damages. We observed an increase in alpha diversity consequent to tree uprooting, an increase that was driven by wood-decay fungi and likely depends on the increase of dead plant material in the area. Besides saprotrophic fungi, specific mycorrhizal taxa seemed to respond to the plant succession, which involved the increase of grasses and the consequent shift in plant symbionts. From a mycological point of view, the investigated ecosystem resulted to be resilient, rather than resistant (insensitive) to the disturbance. We do not have enough data to infer the outcome of the above-mentioned succession, but we argue that the described fungal community is complete in its trophic components, plastic and not affected by dominance of few species, and that could support the repopulation of the habitat. Any naturalistic forestry management activity, which will promote this natural trend, will certainly favor the resilience of these forest formations to extreme events exacerbated by the climate changes underway, especially with regard to drought and windstorms (Ciancio and Nocentini, 2011). Future development of this approach would arguably require monitoring of the fungal community over time, which would help to identify when adverse conditions take place, and which actors are at play before and after a disturbance.

## Data availability statement

The data presented in the study are deposited in the NCBI database, accession number PRJNA1009257.

## Author contributions

FV: formal analysis, writing–original draft, and investigation. AV: validation and data curation. RD: Resources. GDR: conceptualization and funding acquisition. AM: conceptualization, writing–original draft, and supervision. All authors contributed to the article and approved the submitted version.

## Footnotes

^1^ https://CRAN.R-project.org/package=ggridges

^2^ https://github.com/Joseph7e/Assign-Taxonomy-with-BLAST.git
