# Supplementary figures and images for: Responses of a soil fungal community to severe windstorm damages in an old silver fir stand
Source: Front Microbiol. 2023 Nov 10;14:1246874. doi: 10.3389/fmicb.2023.1246874 (PMC10668432; doi:10.3389/fmicb.2023.1246874)

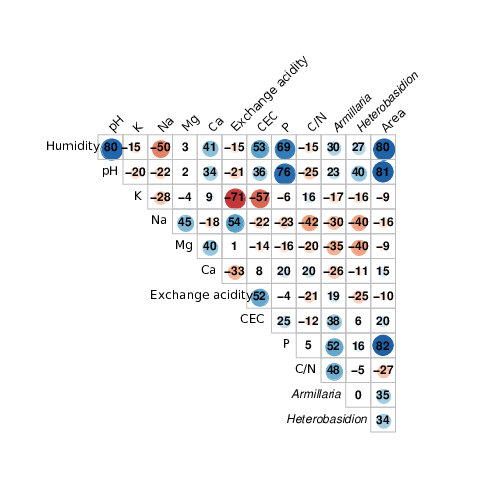

Supplement: Supplementary file 1 [file Image_1.TIFF]

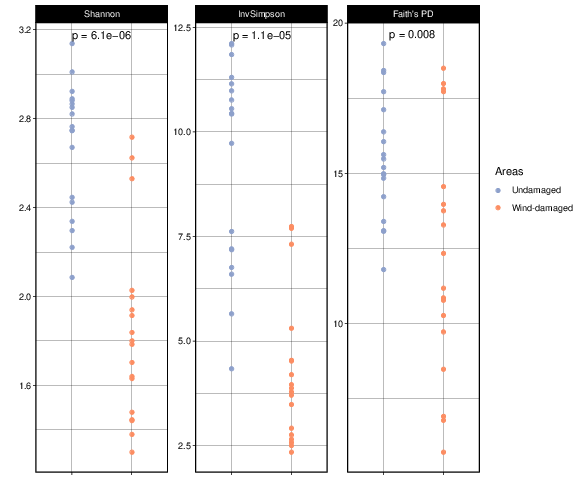

Supplement: Supplementary file 2 [file Image_2.TIFF]

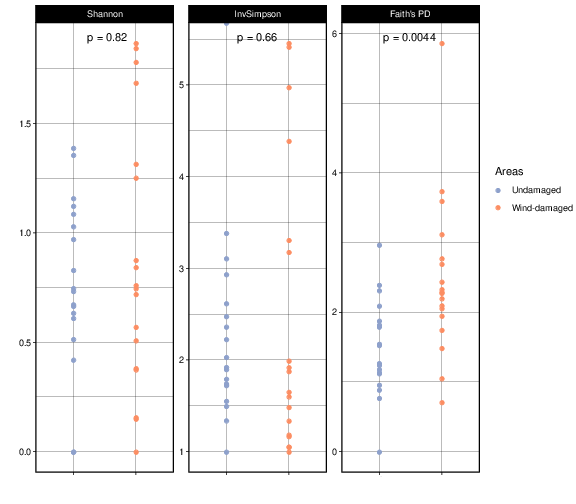

Supplement: Supplementary file 3 [file Image_3.TIFF]

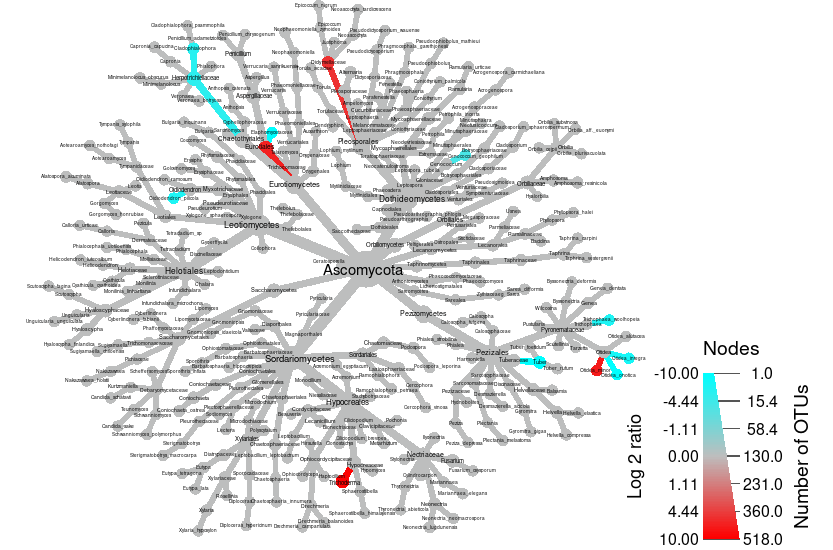

Supplement: Supplementary file 4 [file Image_4.PNG]

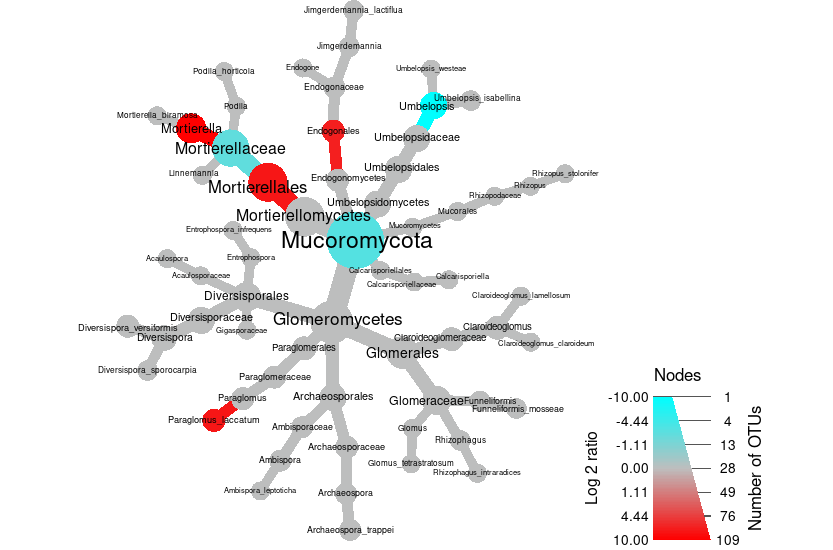

Supplement: Supplementary file 5 [file Image_5.PNG]

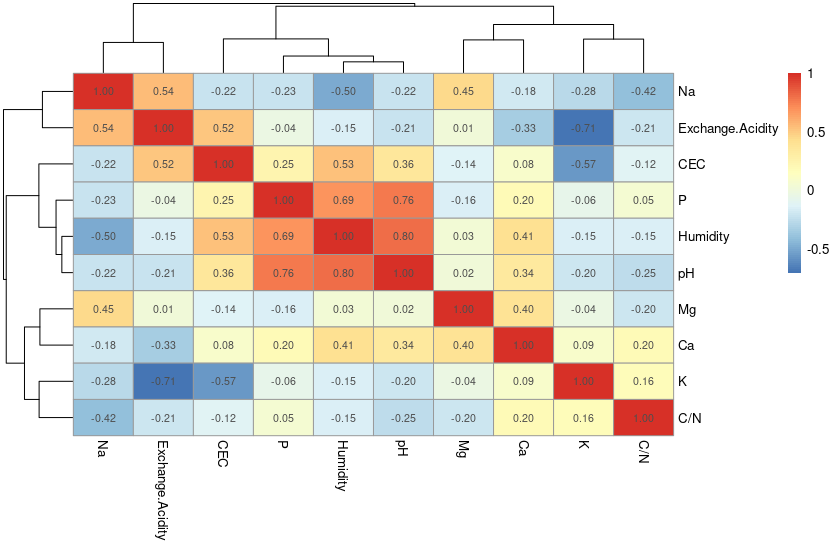

Supplement: Supplementary file 6 [file Image_6.PNG]

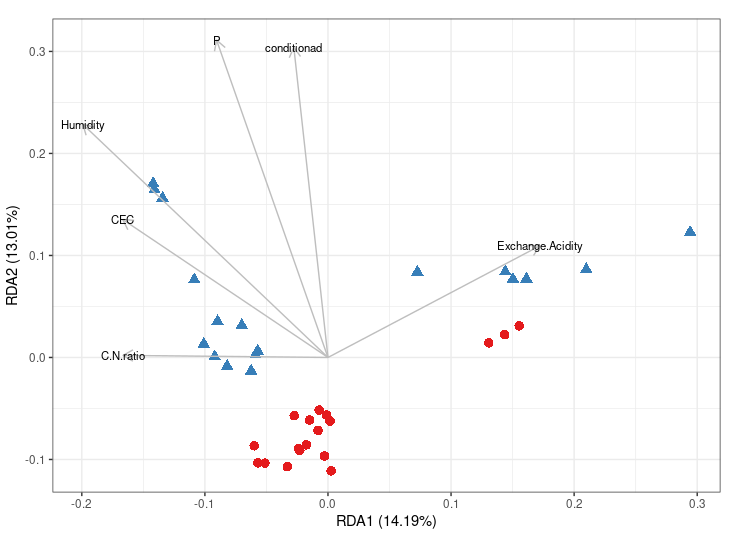

Supplement: Supplementary file 7 [file Image_7.PNG]
